# Long-term survival following serial pulmonary metastasectomies for uterine leiomyosarcoma: a case report

**DOI:** 10.1186/s44215-025-00231-4

**Published:** 2025-11-11

**Authors:** Ken Kodama, Toru Momozane, Hiroshi Takehara, Kazuaki Sato

**Affiliations:** 1 Department of Thoracic Surgery, Yao Municipal Hospital, 1-3-1 Ryuge-Cho, Yao City, Osaka, 581-0069 Japan; 2 Department of Pathology, Yao Municipal Hospital, Osaka, Japan

**Keywords:** Uterine leiomyosarcoma, Multiple pulmonary metastases, Pneumothorax, Surgical treatments, Long-term survival

## Abstract

**Background:**

In patients with uterine leiomyosarcoma and multiple pulmonary metastases complicated by pneumothorax during chemotherapy, repeated metastasectomy may contribute to long-term survival.

**Case presentation:**

A 45-year-old woman underwent total hysterectomy for uterine leiomyosarcoma. She subsequently developed multiple pulmonary metastases and was treated with gemcitabine plus docetaxel (GD) chemotherapy. During the course of chemotherapy, she developed a pneumothorax, possibly as a consequence of tumor necrosis induced by treatment. Due to a rapidly declining in the performance status (PS), right lower lobectomy was performed to manage the pneumothorax and reduce the tumor burden. GD chemotherapy was resumed postoperatively but discontinued after a total of 14 cycles due to adverse events. As anticipated, the pulmonary metastases regrew. However, no evidence of extrathoracic disease was identified, and her respiratory function was deemed sufficient for surgery. She subsequently underwent one-stage partial bilateral lung resections, during which a total of 12 metastatic nodules were removed. Two years later, two additional metastatic lesions were resected. Since that time, 5 years and 8 months have passed without any recurrence or additional treatment. At the time of reporting, the patient had remained disease-free, 16 years after the initial hysterectomy, with PS of 0.

**Conclusion:**

Uterine leiomyosarcoma is an aggressive tumor; however, in selected cases, long-term survival may be achieved through multimodal treatment approaches, including surgical resection of metastatic lesions.

## Introduction

Uterine leiomyosarcoma (uLMS) is an aggressive tumor that can show resistance to standard therapy, making it challenging to treat patients, as evidenced by high rates of both recurrence and progression [1]. In patients whose solitary pulmonary metastasis is present synchronously without a disease-free interval, curative-intent resection of both the primary uLMS and lung lesion has been associated with improved overall survival [2]. In the study by Burt et al. [3] on repeated and aggressive pulmonary metastasectomies in 31 LMS patients including 13 with uLMS, the 5-year survival rate was 52%. In their multivariate analyses, the disease-free interval (DFI) was significantly correlated with a poor prognosis. However, no significant correlation was found between survival and the number of resected tumors. In the 31 patients undergoing metastasectomy who they analyzed, all had five or fewer metastases, and the majority (87%) had two or fewer. Notably, resection involving more than six lesions have rarely been reported.

A pneumothorax secondary to necrosis or cystic degeneration of metastatic lesions due to aggressive tumor growth or antineoplastic therapy is an uncommon but serious complication [4]. Long-term survival in such cases has also rarely been reported.

We report a uLMS patient with multiple pulmonary metastases which involved pneumothorax who has been doing well for more than 5 years since the final metastasectomy.

## Case presentation

**Fig. 1:**
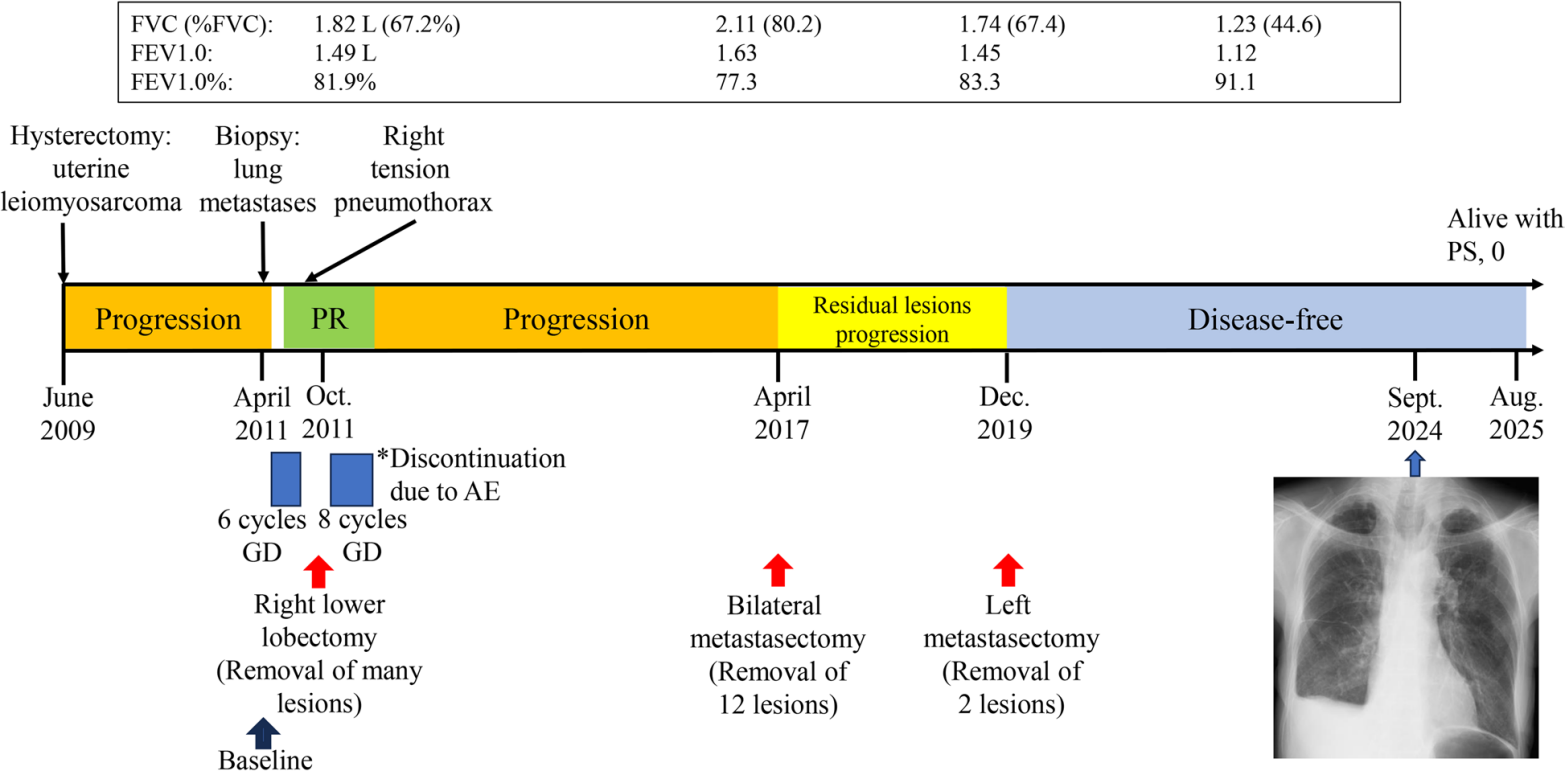
Chronological summary of disease progression and interventions in a patient with uterine leiomyosarcoma. FVC, functional vital capacity; FEV1.0, forced expiratory volume in the 1 st second; PR, partial response; GD, gemcitabine plus docetaxel

A 45-year-old woman presented with low back pain in June 2009 and visited a local hospital. Figure 1 illustrates the timeline of her clinical course. She was diagnosed with uterine fibroids and subsequently underwent a simple total hysterectomy. However, postoperative histopathological review unexpectedly led to a diagnosis of uLMS. The tumor cells were immunoreactive for CD44, CD34, estrogen receptor (ER), and progesterone receptor (PgR), but negative for AE1/AE3. In addition, < 10–20% of tumor cells overexpressed p53, and the MIB1 (Ki67) index was less than 40%.

**Fig. 2:**
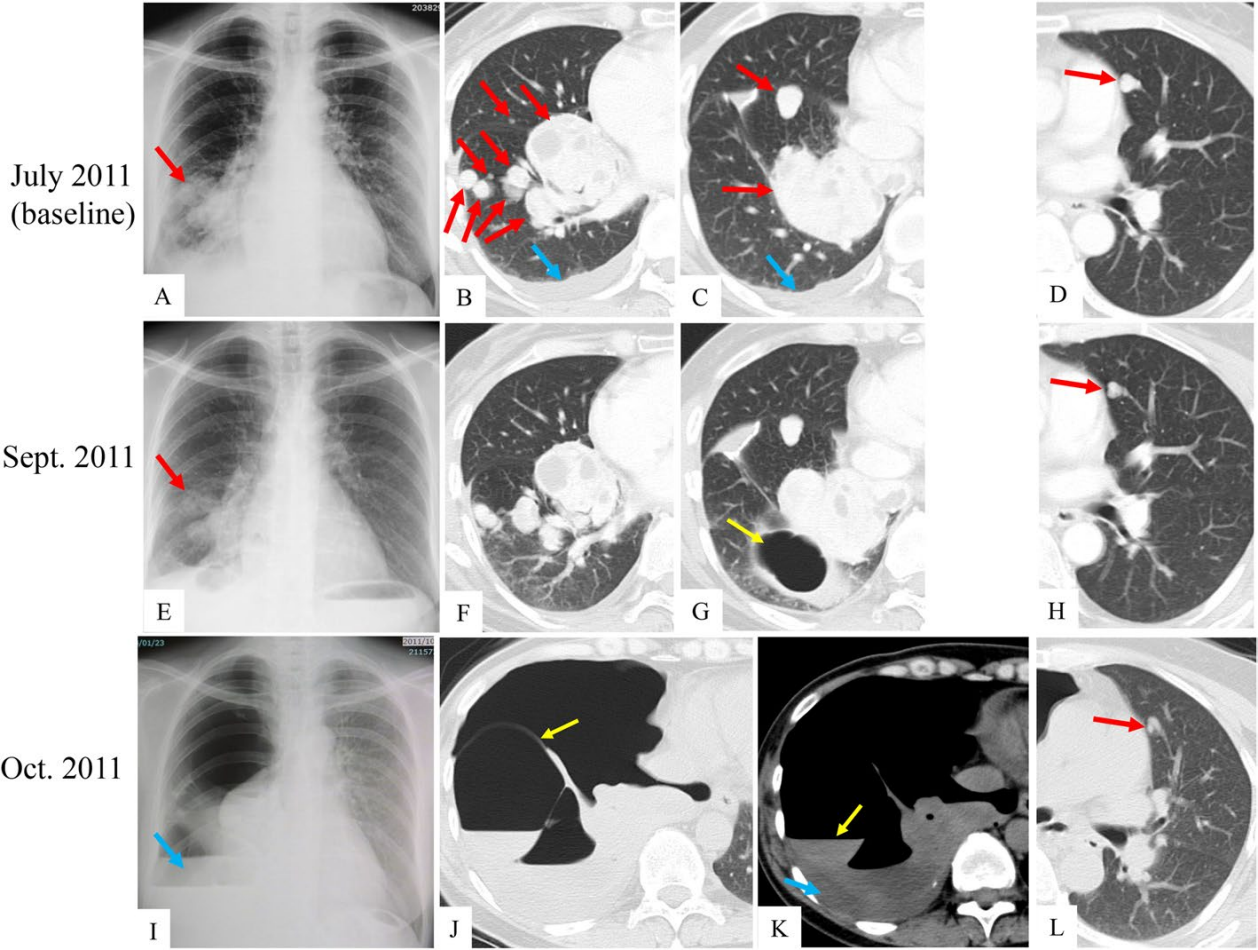
Representative chest radiography and CT images at baseline (**A**–**D**), during chemotherapy (**E**–**H**), and after the onset of pneumothorax with mediastinal shift to the contralateral side (**I**–**L**). Red arrows indicate metastatic tumors; blue arrows, right pleural effusion; yellow arrows, a giant cyst that appeared in the bottom of the basal segment during treatment

In October 2010, a routine medical checkup revealed abnormal shadows in the bilateral lungs (DFI, 14 months). After histologic confirmation of metastatic uLMS, the patient was referred to our oncology department in May 2011 for chemotherapy. Her baseline images showed bilateral pulmonary metastases and right pleural effusion (Fig. 2A-D). Between June and September 2011, she underwent six cycles of gemcitabine plus docetaxel (GD) chemotherapy resulting in a decrease in tumor size (Fig. 2E–H). On October 4, 2011, she presented with acute respiratory distress even at rest and was diagnosed with right pneumothorax based on a chest radiograph and CT (Fig. 2I-L). The tumor showed a heterogenous internal structure. She underwent chest tube drainage. Cytology of pleural effusion was negative. At that time, her Eastern Cooperative Oncology Group performance status (ECOG-PS) was 3. Pulmonary function tests performed after drainage showed values indicating sufficient tolerance for resection of the affected right lower lobe (Fig. 1).

**Fig. 3:**
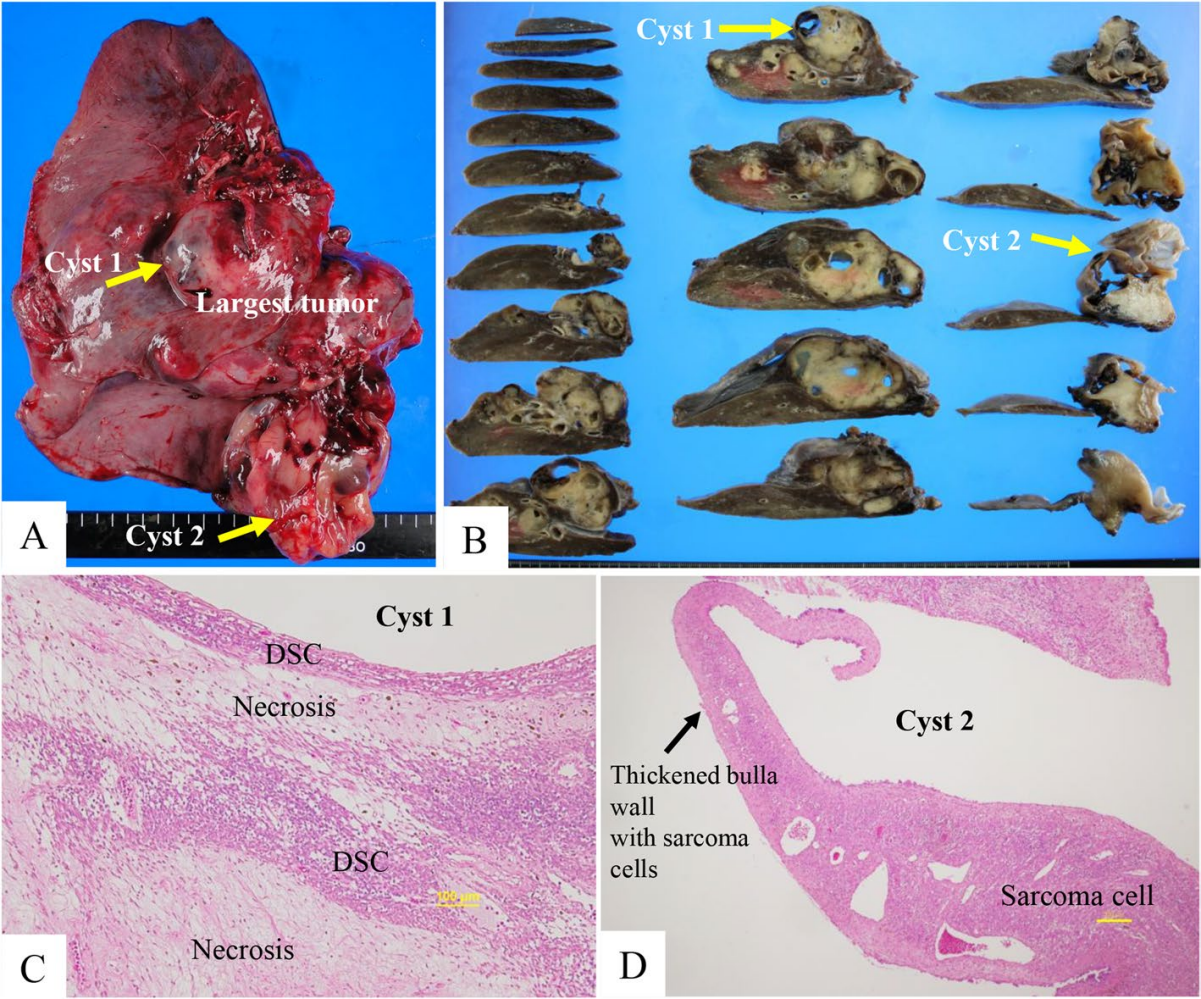
Freshly resected specimen (**A**), cut surface after formalin fixation (**B**), and histopathological findings (**C**, **D**) (hematoxylin and eosin stain). DSC, degenerated sarcoma cells

On October 21, 2011, the patient underwent right lower lobectomy to manage the pneumothorax and reduce the tumor burden. Grossly, multiple solid tumors of various sizes with cystic degeneration were observed in the resected lung, with a maximum diameter of 61 × 35 mm (Fig. 3A). Mucinous (myxoid) degeneration and cysts of varying sizes were present within the tumor (Fig. 3B). Histopathologically, the tumor was diagnosed as LMS. As shown in Fig. 3C, degenerated sarcoma cells (DSC) and area of necrosis were observed adjacent to the cystic cavity, suggesting a therapeutic effect of chemotherapy (viable tumor as 1/3–2/3 of tumor bed). Figure 3D showed the wall of the giant cyst with infiltrated sarcoma cells at the bottom of the basal segment. Eight courses of postoperative GD chemotherapy were administered from December 2011 until May 2012. Thereafter, chemotherapy was discontinued due to severe edema as an adverse event (AE).

**Fig. 4:**
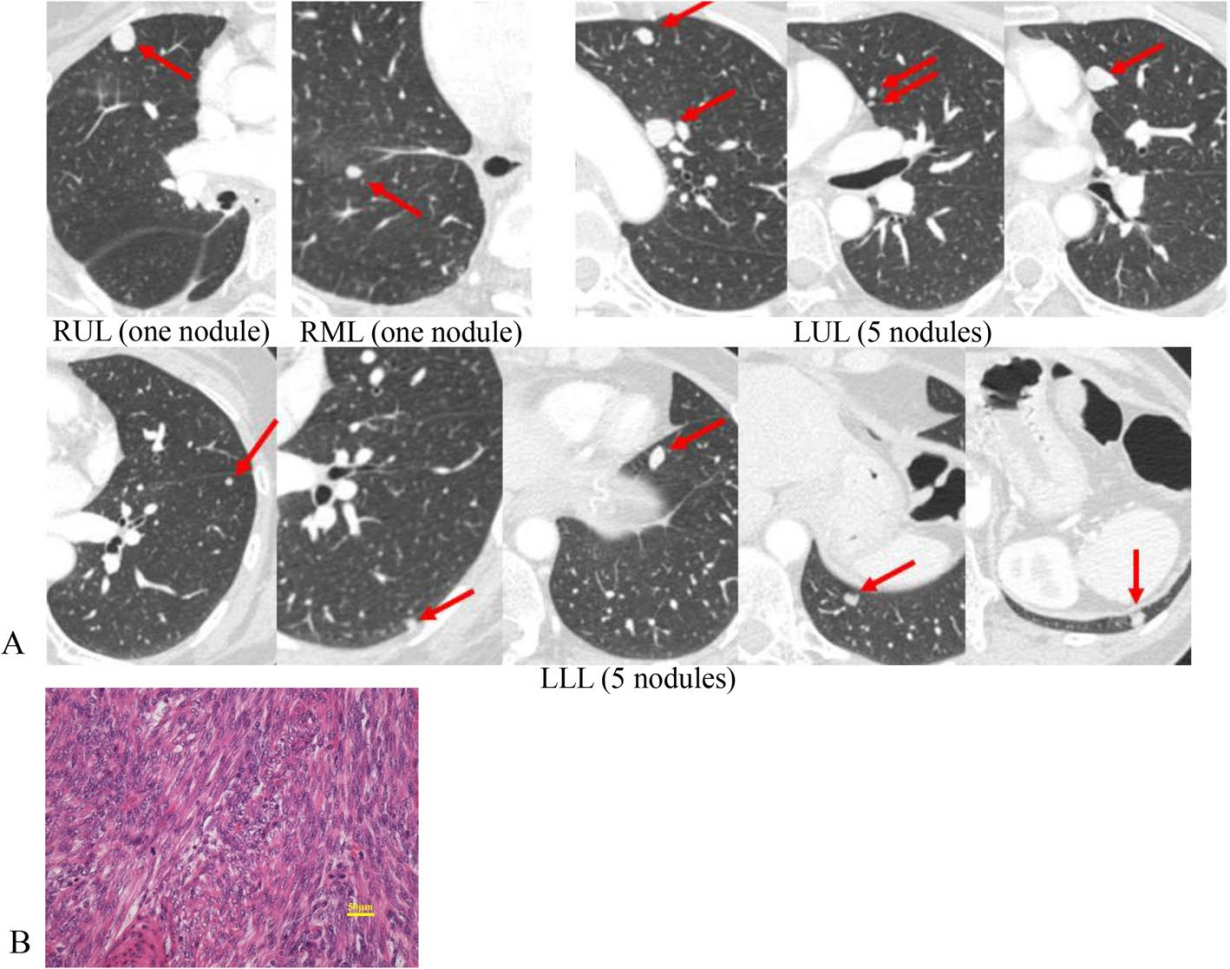
Chest CT images showing 12 lung metastases (red arrows) before the second metastasectomy (**A**), and histopathological finding of the resected specimen from the third metastasectomy (**B**), demonstrating a typical appearance of leiomyosarcoma unaffected by chemotherapy (hematoxylin and eosin stain). RUL, right upper lobe; RML, right middle lobe; LUL, left upper lobe; LLL, left lower lobe

CT of the chest in November 2016 showed a total of 12 nodular shadows, all with a clinical diagnosis of lung metastasis (Fig. 4A). At the second metastasectomy in April 2017, those metastases were removed via one-stage bilateral thoracotomy.

The patient underwent a third metastasectomy for two new metastases in left segment 3 and 8 in December 2019. The histologic features were consistent with metastasis of uLMS (Fig. 4B). Microsatellite instability (MSI) was not detected in the resected specimen.

Figure 1 shows the results of pulmonary function tests conducted before each lung resection and one year after the final resection. At present, 16 years after resection of the primary uterine tumor, and 5 years and 8 months after the final metastasectomy, the patient remains alive with no recurrence or respiratory symptom, and has ECOG-PS of 0.

## Discussion

In the present patient, postoperative histopathological review unexpectedly led to a diagnosis of grade 1, uLMS, but not uterine fibroid, based on the French Federation of Cancer Centers Sarcoma Group (FNCLCC) soft tissue sarcoma grading system. The grade is the sum of the scores allocated for three major histologic criteria: tumor differentiation, mitotic count, and tumor necrosis in soft tissue sarcoma. Although the tumor grade is considered an independent prognostic factor in the FNCLCC grading system, with grade 3 tumors associated with high-level malignancy and a higher risk of distant metastasis, this system is not considered reliable for uLMS [5]. In uLMS, the diagnosis of sarcoma itself is regarded as an unfavorable prognostic factor, and the FNCLCC grading score is not a valid prognostic indicator. In the present case, despite being classified as grade 1, the patient developed multiple pulmonary metastases. Compared with other uterine malignancies, uLMS has a high propensity for local recurrence and distant metastasis [6]. Although, factors indicating a poor prognosis include a tumor size of more than 5 cm and high mitotic index, they are highly aggressive even with a mitotic count of less than 2/mm^2^ [7]. The lungs are the most common site of distant metastasis. Regarding treatment, only resection of metastases reportedly significantly influenced postmetastasis survival in multivariate analysis [6].

Thirteen pulmonary wedge resections were performed at the second and third metastasectomies. Since 1997, we have consecutively performed intraoperative resection margin lavage cytology in cases of malignant pulmonary tumors undergoing wedge resection [8]. Pulmonary metastases of uLMS exhibit well-defined histological boundaries, allowing gross evaluation of resection margins in most cases. In only one of 13 wedge resections, where gross assessment of the margin was difficult, we confirmed the absence of tumor cells via margin lavage cytology.

A pneumothorax associated with pulmonary metastases is typically considered to indicate a poor prognosis. Once pulmonary metastasis of sarcoma becomes associated with pneumothorax, the frequency of pneumothorax recurrence is very high, being 45.7% at an average of 61 (± 112) days [9]. According to the ERS/EACTS/ESTS clinical practice guidelines [10], surgical treatment is conditionally recommended for patients with secondary spontaneous pneumothorax at high risk of recurrence or those with persistent air leak, taking into account the individual patient background.

We were unable to identify the site of air leakage responsible for the pneumothorax intraoperatively. As an alternative mechanism of pneumothorax associated with metastatic pulmonary tumors, a check-valve mechanism was proposed by Minami et al. [11]. Therefore, we performed histopathological examination of the small airway located between the giant cyst and the sarcoma in the bottom of the right basal segment. However, no evidence of airway stenosis suggestive of a check-valve mechanism contributing to cyst enlargement and pneumothorax was observed.

Dissemination of sarcoma cells into the pleural cavity is considered extremely rare in LMS due to its cellular characteristics [12]. Although this patient presented with both pleural effusion and pneumothorax, pleural fluid cytology was negative. In addition, no lymph node metastasis or vascular invasion was observed. Given these conditions, this patient demonstrated that multidisciplinary management, including prompt surgical intervention, appropriate systemic chemotherapy, and close monitoring, can result in favorable long-term outcomes, even in the presence of treatment-related AE such as pneumothorax.

Garcia et al. [13] reported one unique patient with recurrent pulmonary metastases who responded to 1 st and 2nd line chemotherapies and sequential hormone therapies using megestrol and anastrozole, in combination with repeated metastasectomies. This exceptional patient survived for more than 9 years after her initial diagnosis. Our patient was also very similar to the one reported by them. Both responded to chemotherapy and it might suggest that micrometastases throughout the body were well controlled. They also were ER/PgR-positive. However, MSI was not detected in our patient. Therefore, if our patient shows recurrence in the future, it may be worthwhile to try aromatase inhibitors (AIs) such as anastrozole or letrozole, although published data regarding the efficacy of AIs for this subtype of uLMS are limited [14].

In conclusion, we reported a uLMS patient. Following total hysterectomy, the patient developed multiple pulmonary metastases, which responded to chemotherapy. However, during treatment, she developed pneumothorax, leading to right lower lobectomy including the responsible metastatic lesion. Subsequently, she underwent two additional metastasectomies for bilateral pulmonary metastases. In total, more than 20 metastatic lesions were resected through three metastasectomies. As a result, the patient currently remains disease-free 16 years after the initial hysterectomy. Accumulation of such exceptional cases and further investigation to identify predictors of the treatment response are warranted.

## Data Availability

The data that support the findings of this study are available from the corresponding author upon reasonable request.

## Abbreviations

uLMS: Uterine leiomyosarcoma
DFI: Disease-free interval
ER: Estrogen receptor
PgR: Progesterone receptor
CT: Computed tomography
GD: Gemcitabine plus docetaxel
ECOG-PS: Eastern Cooperative Oncology Group performance status
DSC: Degenerated sarcoma cells
MSI: Microsatellite instability
FNCLCC: French Federation of Cancer Centers Sarcoma Group
AIs: Aromatase inhibitors
